# Reversible Regulation of Polar Gas Molecules by Azobenzene-Based Photoswitchable Metal–Organic Framework Thin Films

**DOI:** 10.3390/molecules28020877

**Published:** 2023-01-15

**Authors:** Xuan Chen, Siyu Fang, Ping Xue, Jiming Huang, Mi Tang, Zhengbang Wang

**Affiliations:** 1 Ministry of Education Key Laboratory for the Green Preparation and Application of Functional Materials, Hubei Key Laboratory of Polymer Materials, Collaborative Innovation Center for Advanced Organic Chemical Materials Co-Constructed by the Province and Ministry, School of Materials Science and Engineering, Hubei University, Wuhan 430062, China; 2 School of Pharmacy, Hubei University of Science and Technology, Xianning 437100, China; 3 School of Material and Chemical Engineering, Tongren University, Tongren 554300, China

**Keywords:** metal–organic frameworks, azobenzene, photoswitchable, gas separation

## Abstract

The development of tunable molecule separation membranes requires materials with remote controllability and ultra-high separation capability. In this paper, a novel photoswitchable metal organic framework (MOF) thin film (Cu_2_(AzoBPDC)_2_) was prepared by liquid phase epitaxial layer-by-layer assembly to realize the reversible remote-controlled switching. The azobenzene side groups in the Cu_2_(AzoBPDC)_2_ thin film showed excellent reversible photoswitching performance under UV (365 nm) and Vis (450 nm) irradiation, achieving the remote-controlled mode of the diffusion flux of polar gas molecules in the MOF thin film.

## 1. Introduction

Due to its remote-controllable physical and chemical properties, smart materials have been widely taken into consideration and enabled a variety of specific advanced applications [1,2,3,4]. Light stimulus enables the on/off function of the photoswitchable materials in remote-controlled mode in comparison with other external stimuli (such as temperature, electric field, pH value) and is considered to be the most-prospective technology. In recent years, various photoswitchable molecules have been reported [5,6,7,8], and since the *cis*-form azobenzene was first discovered under UV irradiation [9], azobenzene has become the most widely studied photoswitchable molecule between *trans* and *cis* [10,11,12]. As a smart material, photoswitchable azobenzene-containing metal organic frameworks (MOFs) with periodic nano-porous structures [13,14,15], high specific areas, and structural designability are particular promising and interesting. The physical and chemical properties and pore structure of photoswitchable azobenzene-containing MOFs could be remotely controlled by changing the external light stimulus [16,17]. Based on this, it can be applied in gas separation, such as adjusting the size of the pore by light stimulus, so as to realize the efficient separation of gas or liquid mixtures. In this context, the photoswitchable azobenzene-containing MOFs hold a substantial potential application in separation, since they offer a low-cost, energy-saving, and environmentally friendly alternative to traditional separation technology [5,18].

To obtain the photoswitchable azobenzene-containing MOFs, azobenzene units are usually integrated (or filled) into the MOF structure as a functional group (or a guest molecule) [19,20,21]. However, the filling of azobenzene molecules into the MOF channels has certain requirements on the pore size of the MOFs [2,22], and isomerization may be hampered due to the restricted space; however, azobenzene, as the side group of the MOFs, can overcome this difficulty. The introduced azobenzene units as dangling groups have enough free space for the *trans*-to-*cis* conversion. In addition, there are only a few cases based on photoswitchable MOFs, especially photoswitchable MOF thin films. Therefore, it is of great significance to design and prepare novel photoswitchable MOF thin films to enrich their structures and properties.

**Scheme 1:**
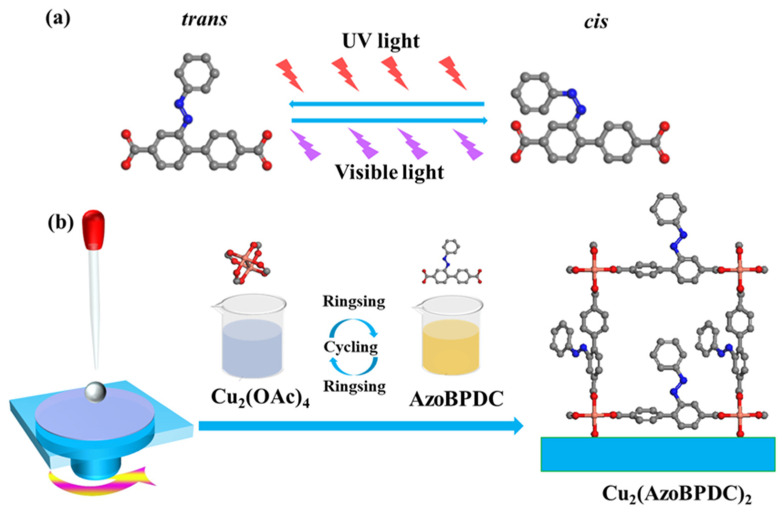
Schematic illustration of the switching of the AzoBPDC linker (**a**) and the preparation of the corresponding Cu_2_(AzoBPDC)_2_ thin film (**b**).

Here, we report a novel photoswitchable MOF thin film with azobenzene units as the side groups. The Cu_2_(AzoBPDC)_2_ thin film was prepared by the liquid phase epitaxial layer-by-layer assembly technique [23] using AzoBPDC and copper acetate as precursors, as shown in Scheme 1. The micropores’ structure and the surface properties of the thin films can be adjusted by illuminating them with different wavelengths of light based on the switching of the azobenzene side groups, and then, the diffusion flux of the gas molecules in the thin film can be controlled remotely.

## 2. Results and Discussion

**Figure 1:**
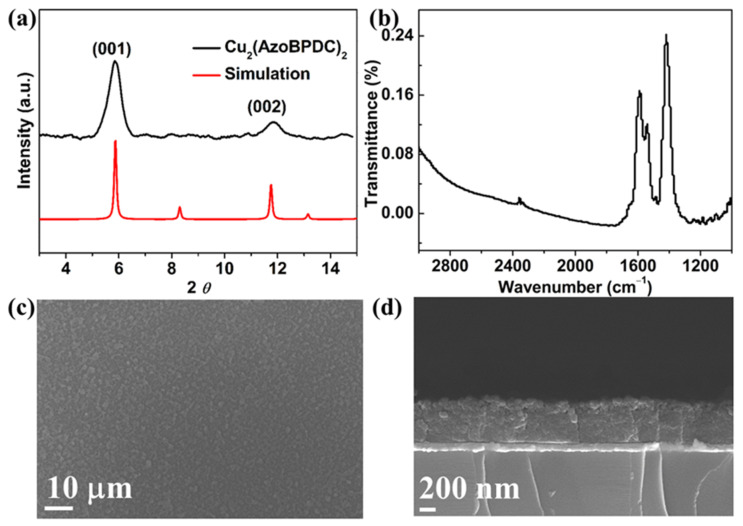
(**a**) XRD patterns of Cu_2_(AzoBPDC)_2_ film; (**b**) IRRAS of Cu_2_(AzoBPDC)_2_ film; SEM images of Cu_2_(AzoBPDC)_2_ thin film (**c**) surface; (**d**) cross-section.

Firstly, to explore the structure of the Cu_2_(AzoBPDC)_2_ thin film, XRD and IRRAS were employed. As shown in Figure 1a, the prepared Cu_2_(AzoBPDC)_2_ thin film exhibited a highly oriented and crystallinity structure with diffraction peaks at 5.8° and 11.8°, corresponding to the crystal planes of (001) and (002), respectively, exhibiting a similar SURMOF-2 structure based on a regular packing of (Cu^++^)_2_-carboxylate paddle-wheel planes with a P4 symmetry, a series of isoreticular MOF structures described in the previous work [24]. The results were consistent with the simulated data, indicating the successful synthesis of the MOF film. As can be seen from IRRAS (Figure 1b), there were no bands between 1800 and 1600 cm^−1^, demonstrating that no free carboxyl group existed in the MOF film. In addition, the strong characteristic bands in the range of 1600–1400 cm^−1^ can be attributed to the symmetric stretching vibration of carboxylate [25]. The IRRAS analysis showed that the AzoBPDC functional unit formed an excellent coordination with the copper ions, reconfirming the successful acquisition of the Cu_2_(AzoBPDC)_2_ thin film.

To the best of our knowledge, the thickness and surface roughness of thin films have a strong impact on the quality and optical and gas separation properties of thin films. Therefore, the surface and cross-section of the prepared thin film were subsequently investigated by FE-SEM. As shown in Figure 1c, the surface SEM image of the Cu_2_(AzoBPDC)_2_ thin film displayed a relatively flat and evenly distributed surface structure. Note that the uniformity and high flatness of the film were more conducive to the subsequent study of the optical and gas separation performance. From the cross-section of the Cu_2_(AzoBPDC)_2_ thin film (Figure 1d), it can be observed that the prepared thin film was very dense with a thickness of about 400 nm, further proving that the prepared thin film was ultra-high quality and could meet the follow-up research.

**Figure 2:**
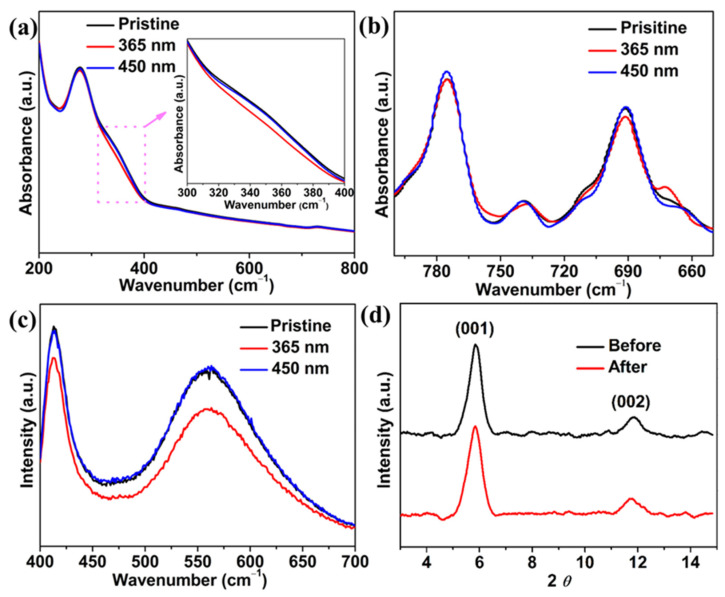
(**a**) UV–Vis (**b**) IRRAS, and (**c**) PL emission spectra of Cu_2_(AzoBPDC)_2_ film (pristine (black); after UV irradiation (red,); after visible light irradiation (blue)); (**d**) XRD patterns of Cu_2_(AzoBPDC)_2_ film before and after UV–Vis irradiation performed once.

To investigate the photoswitching of the azobenzene moieties in the Cu_2_(AzoBPDC)_2_ thin film, UV–Vis, IRRAS, and PL were performed. In Figure 2a, it can be observed that the azobenzene π-π* band (near 350 nm) significantly decreased under the UV irradiation with a Xe lamp with a 365 nm wavelength. Afterwards, the thin film was illuminated with visible light (a Xe lamp with a 450 nm wavelength), and the azobenzene π-π* band (near 350 nm) increased and was restored to the initial state. These results are consistent with a previous report [21]. These intensity variations in the UV–Vis spectra clearly show the *trans*-to-*cis* isomerization caused by the UV irradiation and the opposite change by the visible light irradiation. Meanwhile, IRRAS was used to further characterize the photo response of the MOF thin film by irradiation with UV and visible light. As shown in Figure 2b, upon irradiation with UV light, the intensities of the *trans* azobenzene bands at 775 and 690 cm^−1^ decreased, while that of the *cis* azobenzene band at 672 cm^−1^ increased. [26] Additionally, the *cis* band decreased and *trans* band increased under visible light irradiation, further confirming the photoswitching properties of the Cu_2_(AzoBPDC)_2_ thin film. Furthermore, the reversible changes of the vibration band at around 738 cm^−1^ could be also due to the reversible photoswitching of the azobenzene bands. The PL spectrum provided additional evidence of the light response of the Cu_2_(AzoBPDC)_2_ thin film, as shown in Figure 2c. The PL emission intensity of the thin film decreased after irradiation with UV, indicating that the excitation photons were reduced. Then, when exposed to visible light, the PL emission intensity can be enhanced and restored to the pristine state, with an increase in the excitation photons, which is consistent with the results of the UV–Vis spectra. The above optical experiments proved the successful synthesis of the photoswitched MOF film, and the photo response group (azobenzene) could smoothly carry out photoisomerization in the MOF channels. Moreover, the crystallinity before and after the photo response was measured to verify the stability of the film. As depicted in Figure 2d, the XRD of the thin film before and after UV–Vis irradiation had no obvious change, showing that the structure of the Cu_2_(AzoBPDC)_2_-MOF will not be destroyed by the process of the photo response.

**Figure 3:**
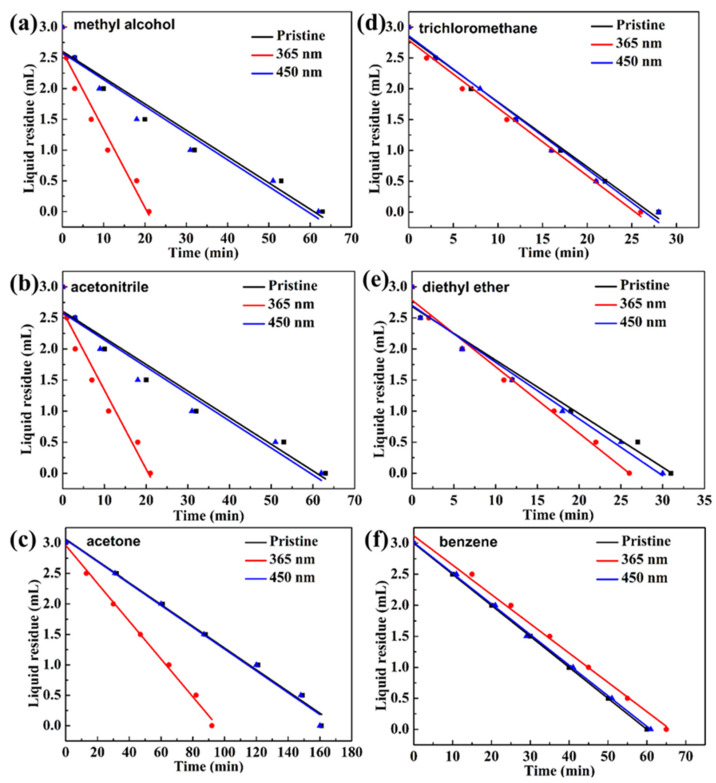
Diffusion flux of polar (**a**–**c**) and non-polar gas molecules (**d**–**f**) (the flow rate of methanol (**a**); acetonitrile (**b**); acetone (**c**); trichloromethane (**d**); diethyl ether (**e**), and benzene (**f**)) (pristine (black); after UV irradiation (red); after visible light irradiation (blue)).

The photoisomerization of the azobenzene side group usually changes its dipole moment and, thus, the polarity of the MOF (note that the dipole moments of *cis* and *trans* azobenzene are about 3.2 and 0 D, respectively [20]). One hypothesis may explain the different effects of the photoswitching on the diffusion flux of the gas molecules, namely the different interaction between the gas molecules and frameworks, depending on the polarity of the gas molecules. To test this hypothesis, the gas separation performance of the Cu_2_(AzoBPDC)_2_ film in the *trans* and *cis* states was investigated. Three polar molecules (methanol, acetone, and acetonitrile) and three non-polar molecules (ether, trichloromethane, and benzene) were selected to test their diffusion flux in the Cu_2_(AzoBPDC)_2_ film. As shown in Figure 3a–c, when the sample is irradiated by UV, the azobenzene side group changes from *trans* to *cis*, resulting in the increased polarity of the MOF thin film. Consequently, as the polar gas molecules pass through the MOF film, they quickly diffuse into the Cu_2_(AzoBPDC)_2_ film due to the dipole interaction, thus increasing the diffusion flux of the polar molecules. Then, the isomerism of the azobenzene side group reverses under visible light irradiation, and the gas diffusion flux returns to the initial state. When the non-polar gas molecules pass through the UV-irradiated MOF film, they generate a weaker dipole interaction, which results in the diffusion flux of the non-polar molecules not changing significantly from the pristine state (Figure 3e,f). It can be concluded that the diffusion flux of the polar molecules through the MOF film significantly varies before and after the photo response of the azobenzene side groups, while that of the non-polar molecules does not. Additionally, by comparing the diffusion flux of gas molecules under different conditions (Table 1), it was found that the diffusion flux was closely related to the polarity of the gas molecules, that is the greater the polarity of the molecules, the faster the diffusion flux of the MOF film with the *cis* states. Therefore, the diffusion flux of the polar gas of the prepared MOF film can be remotely controlled by irradiation and has a bright application prospect in the field of gas separation as a smart material. 

## 3. Materials and Methods

### 3.1. Materials’ Preparations

The chemicals were purchased from Aladdin or Sinopharm Chemical Regents Co., Ltd. (Shanghai, China) and used without further purification. The synthesis of AzoBPDC was based on a previous report [1]. The details of the synthesis (Figure S1) and ^1^H NMR spectrum (Figure S2) of AzoBPDC are shown in the Supporting Information.

The functionalization of the substrate: Firstly, the quartz glass or Al_2_O_3_ substrate was soaked in anhydrous ethanol for 10 min, then washed with anhydrous ethanol three times, dried, and placed in the ultraviolet irradiation instrument for 10 min before the preparation of MOF thin films. For the gold substrate, it was soaked in a 16-mercaptohexadecanoic acid (MHDA) solution for 12 h to form the self-assembled monolayer. Subsequently, it was washed with anhydrous ethanol three times and then dried before the preparation of the MOF thin films.

The preparation of the Cu_2_(AzoBPDC)_2_ thin films: Typically, the substrate was first fixed on the sample support, and then, the copper acetate monohydrate (Cu(OAc)_2_·H_2_O: 1 mM) ethanolic solution and AzoBPDC (2-phenyldiazo-4,4’-biphenyl dicarboxylic acid, 0.2 mM) ethanolic solution were sequentially added dropwise into the rotating functionalized substrate (500 rad/min) for 15 s. Between each step, the substrate was rinsed with pure ethanol for 10 s to remove residual reactants. A continuous cycling (60 cycles) of the above steps allowed growing a high-quality Cu_2_(AzoBPDC)_2_ thin film on the substrate. Before the further experiments and characterizations, all of the MOF thin films were activated in an oven at 60 °C for 24 h to remove the residual solvent.

### 3.2. Characterizations

The structure and morphology of all the samples were characterized by X-ray diffraction (XRD), Fourier transform infrared (FT-IR) spectroscopy, and field emission scanning electron microscopy (FE-SEM). The photoswitching of the Cu_2_(AzoBPDC)_2_ thin film was explored with the ultraviolet–visible (UV–Vis) absorption spectrum, infrared reflection absorption spectroscopy (IRRAS), and steady/transient state fluorescence spectrometry (PL). The detailed instrument model and test methods are documented in the Supporting Information.

### 3.3. Measurement of Diffusion Flux of Gas Molecules

The diffusion flux of the gas molecules was measured by a home-made device, as shown in Figure S3, which consisted of an upper cavity and a lower cavity. The detailed method was as follows: firstly, the organic solvent was injected into the lower cavity, and then, the prepared Cu_2_(AzoBPDC)_2_ thin film (Al_2_O_3_ substrate) was placed in the middle of the upper and lower cavity. Subsequently, the switch valve at the top of the device was connected to a drying tower, a pressure test table, a control valve, and a multi-purpose vacuum pump in turn. Finally, the vacuum pump was opened and the pressure kept at 0.08 Mpa. Then, the control valve was slowly opened to stabilize the pressure of the pressure gauge at 0.08 Mpa. At this time, the solvent in the lower cavity was gradually vaporized and diffused through the prepared Cu_2_(AzoBPDC)_2_ thin film. During this process, the specific value of the remaining solvent could be read out by the scale of the lower cavity.

## 4. Conclusions

To enrich the structure of photoswitchable MOF films, a new azobenzene-based MOF (Cu_2_(AzoBPDC)_2_) film was prepared by the layer-by-layer self-assembly spraying method, and its impact on the diffusion flux of the gas molecules was explored. The photoisomerization of the azobenzene side groups in the MOF film, caused by UV–Vis irradiation, was verified by the UV–Vis, IRRAS, and PL spectra. Diffusion experiments of different gas molecules showed that the effect of the photoswitching of azobenzene in Cu_2_(AzoBPDC)_2_ films on the diffusion flux mainly depends on the polarity of the gas molecules (here, polar molecules). When the azobenzene side groups in MOF channels switch to the *cis* state, the diffusion flux of the polar molecules significantly increases, but that of the non-polar molecules does not change. Furthermore, the diffusion flux of the gas molecules increased to some extent with its polarity. This work not only further enriches the structure of photoswitchable MOF films, but also lays the foundation for the large-scale application of future smart materials.

## Figures and Tables

**Table 1:** Diffusion flux of polar and non-polar gas molecules under different conditions (pristine and under UV–Vis irradiation).

	Methanol (mL/(min·cm^2^)	Acetonitrile (mL/(min·cm^2^)	Acetone (mL/(min·cm^2^)	Trichloromethane (mL/(min·cm^2^)	Diethyl Ether (mL/(min·cm^2^)	Benzene (mL/(min·cm^2^)
Pristine	0.05456	0.05533	0.02268	0.13389	0.10994	0.06369
*Cis*	0.16177	0.16828	0.03949	0.13987	0.13648	0.06019
*Trans*	0.05540	0.05464	0.02280	0.13771	0.11639	0.06294

## Data Availability

Not available.

## Supplementary Materials

The following supporting information can be downloaded at: https://www.mdpi.com/article/10.3390/molecules28020877/s1, Figure S1: Synthesis of AzoBPDC; Figure S2: ^1^H NMR spectrum of AzoBPDC; Figure S3: The device diagram of the gas separation experiment.

## Sample Availability

Samples of the compounds are available from the authors.
