# Supplementary material for: Reversible Regulation of Polar Gas Molecules by Azobenzene-Based Photoswitchable Metal–Organic Framework Thin Films
Source: Molecules. 2023 Jan 15;28(2):877. doi: 10.3390/molecules28020877 (PMC9860582; doi:10.3390/molecules28020877)
Supplement: Supplementary file 1 [file molecules-28-00877-s001.zip › molecules-2099104-supplementary.pdf]

Supplementary Materials

Reversible Regulation of Polar Gas Molecules by Azobenzene-Based Photoswitchable Metal–Organic Framework Thin Films

Xuan Chen [1], Siyu Fang [1], Ping Xue [1,2,*], Jiming Huang [1,3], Mi Tang [1] and Zhengbang Wang [1,3,*]

[1] Ministry of Education Key Laboratory for the Green Preparation and Application of Functional Materials, Hubei Key Laboratory of Polymer Materials, Collaborative Innovation Center for Advanced Organic Chemical Materials Co-Constructed by the Province and Ministry, School of Materials Science and Engineering, Hubei University, Wuhan 430062, China
[2] School of Pharmacy, Hubei University of Science and Technology, Xianning 437100, China
[3] School of Material and Chemical Engineering, Tongren University, Tongren 554300, China
* Correspondence: pingxue@hbust.edu.cn (P.X.); zhengbang.wang@hubu.edu.cn (Z.W.)

Materials' preparation

Detail synthesis of AzoBPDC (Figure S1)

Firstly, 2.0 g (7.4 mmol) of dimethyl biphenyl-4,4′-dicarboxylate was dissolved in 10 mL of concentrated sulfuric acid. Subsequently, the mixed acid (0.51 mL of HNO_3 (65%) dissolved in 3 mL concentrated sulfuric acid) was added into this solution under an ice bath. The mixture was stirred for an additional 15 min, then slowly poured into ice water and continuously stirred. Finally, the white solid (Intermediate 1) obtained by filtration was washed with water and then dried under vacuum. Yield: 82%.

Then, 2.2 g (6.98 mmol) of Intermediate 1, 250 mg of Pd/C (10%), 25 mL of CH_2Cl_2, and 25 mL of methanol were added into a single flask. Subsequently, the mixture was stirred under a hydrogen atmosphere for 18 h. Finally, the mixture was filtered, washed with methanol, and concentrated to obtain Intermediate 2. Yield: 75%.

Next, 0.29 g (1 mmol) of Intermediate 2 was dissolved in 15 mL CH_3COOH. Then, 0.19 g (1.5 mmol) of nitrosobenzene was added into the mixture, and the mixture was stirred at 40℃ for 4 days. Then, the mixture was evaporated to obtain the solid, which was chromatographed with CH_2Cl_2 to afford the orange solid (Intermediate 3). Yield: 52%.

Then, 75 mg (0.2 mmol) of Intermediate 3, 2 mL of KOH (1 M) and 2 mL of THF were added into a single flask, and the mixture was refluxed for 16 h. After cooling to room temperature, the THF was removed by evaporated, and the solution was acidified with HCl (1M). Finally, the orange solid (AzoBPDC) obtained by filtration was washed with water and methanol and then dried under vacuum. Yield: 91%. ^{1}H NMR (**Figure S2**): (400 MHz, DMSO-d_6, δ ppm) 13.17 (bs, 2H), 8.28-8.27 (d, 1H), 8.21-8.19 (m, 1H), 8.06-8.04 (d, 2H), 7.84-7.77 (m, 3H), 7.66-7.64 (d, 2H), 7.59-7.58 (m, 3H).

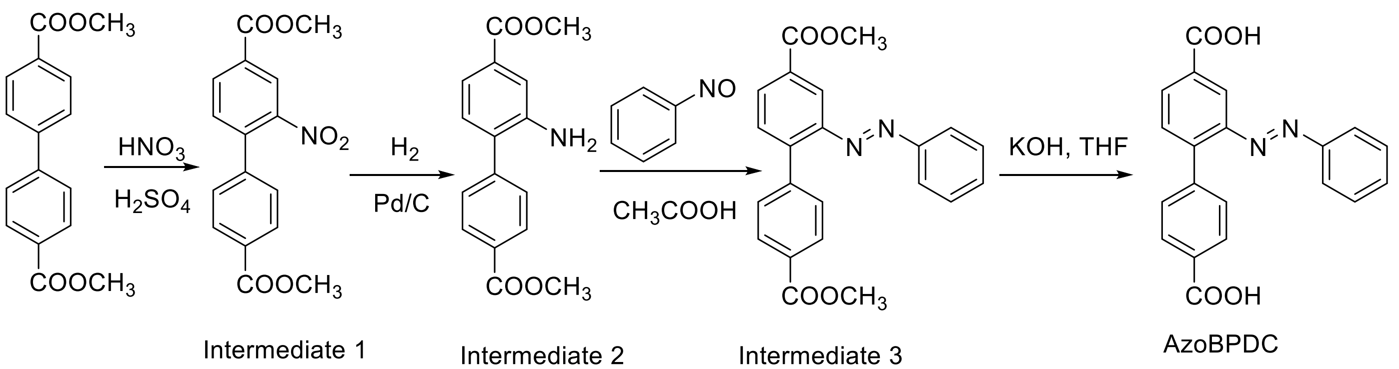

Figure S1. Synthesis of AzoBPDC.

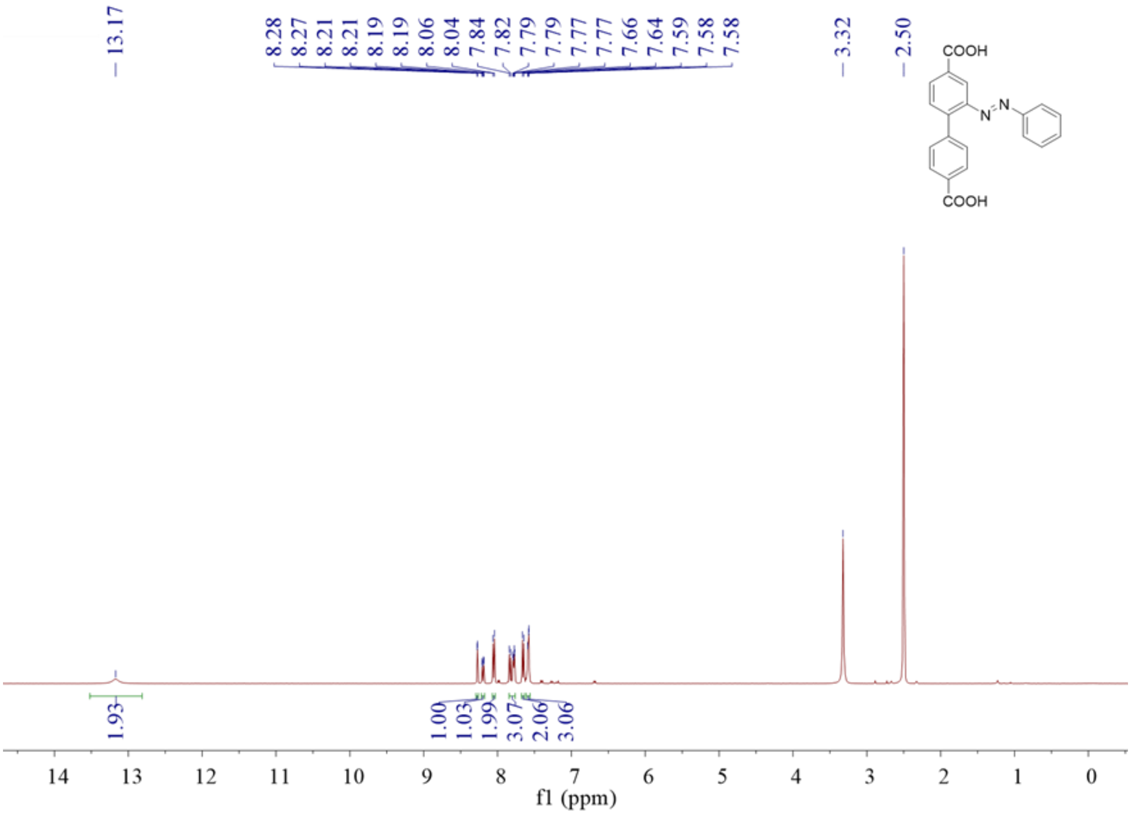

Figure S2. ^{1}H NMR spectrum of AzoBPDC.

Characterization

Characterization was performed by X-ray diffraction (XRD, Bruker D8 with Cu Kα radiation, 40 KV, 40 mA, λ = 1.5418 Å), Fourier transform infrared spectrometry (FT-IR, Nicolet iS50), field emission scanning electron microscopy (FESEM, Sigma 500, Germany), ultraviolet–visible (UV–Vis, UV3600, SHIMADZU, Japan).

In the photo response characterizations and diffusion experiments, the UV–Vis irradiation lasted 30 min. The XRD characterization was as follows: firstly, the MOF thin film was placed in the groove of a quartz sample stage, and the film was padded with plasticine to keep the MOF film at the same height as the top of the groove. Then, the test was carried out (step size: 0.02°, time: 0.5 s, 2θ: 3-15°).

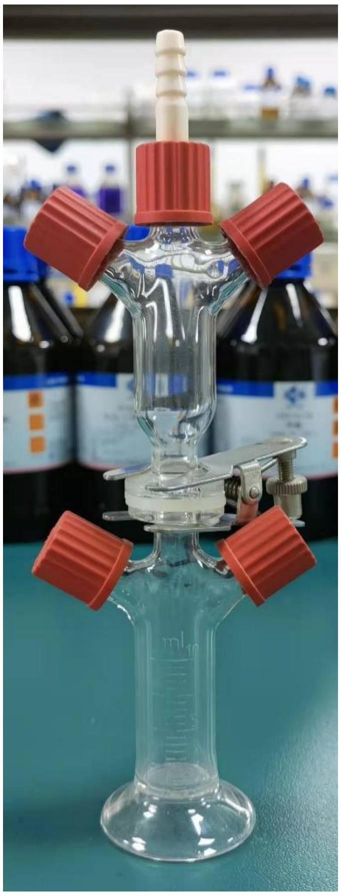

Figure S3. The device diagram of the gas separation experiment.
